# Supplementary material for: Development of EphA2 siRNA-loaded lipid nanoparticles and combination with a small‐molecule histone demethylase inhibitor in prostate cancer cells and tumor spheroids
Source: J Nanobiotechnology. 2021 Mar 8;19:71. doi: 10.1186/s12951-021-00781-z (PMC7938557; doi:10.1186/s12951-021-00781-z)
Supplement: Supplementary file 1 — Additional file 1: Fig. S1. Representative Western blot image showing the basal expression levels of EphA2 protein in LNCaP, DU145, and PC-3 prostate cancer cells. Fig. S2. Full scans of Western blot images corresponding to Fig. 6c, d. Marker bands: 250, 150, 100, 75, 50, 37, 25, 20 kDa. Fig. S3. The effect of siEphA2 complexes co-administered with JIB-04 on cell viability in RWPE-1 (A) and PWR-1E (B) normal human prostate epithelial cell lines (48 h). Data are given as mean ± SD of three measurements. Fig. S4. The morphological changes in PC-3 spheroids after co-treatment of siEphA2 complexes with JIB-04. Scale bar, 1000 µm. Fig. S5. Representative phase-contrast images of PC-3 cells showing the changes in scratch area in response to treatments after 0 h, 24 h, 48 h, and 72 h. MRI Wound Healing Tool plugin of ImageJ was used to automatically draw the scratch area. Scale bar, 1000 µm. [file 12951_2021_781_MOESM1_ESM.docx]

**Additional file 1**

**Development of EphA2 siRNA-loaded lipid nanoparticles and combination with a small-molecule histone demethylase inhibitor in prostate cancer cells and tumor spheroids**

Ezgi Oner^a,b,c^, Mustafa Kotmakci^b^, Anne-Marie Baird^a,d,e^, Steven G. Gray^d,e^, Bilge Debelec Butuner^b^, Emir Bozkurt^f^, Ayse Gulten Kantarci^b,1^, Stephen P. Finn^a,d,g,h,1 *^

*^a^ Department of Histopathology and Morbid Anatomy, Sir Patrick Dun Translational Research Lab, St. James’s Hospital, Dublin, Ireland.*

*^b^ Department of Pharmaceutical Biotechnology, Faculty of Pharmacy, Ege University, Bornova, Izmir, Turkey.*

*^c^ Department of Pharmaceutical Biotechnology, Faculty of Pharmacy, Izmir Katip Celebi University, Balatcik, Izmir, Turkey.*

*^d^ Thoracic Oncology Research Group, Trinity Translational Medicine Institute, St. James’s Hospital, Dublin, Ireland.*

*^e^ Department of Clinical Medicine, Trinity College Dublin, Dublin, Ireland.*

*^f^ Department of Genetics and Bioengineering, Faculty of Engineering, Izmir University of Economics, Balcova, Izmir, Turkey.*

*^g^ Department of Histopathology, Labmed Directorate, St. James’s Hospital, Dublin, Ireland.*

*^h^ Cancer Molecular Diagnostics, Labmed Directorate, St. James’s Hospital, Dublin, Ireland.*

*^1^ Joint senior authorship.*

*Corresponding author: S. P. Finn. E-mail: stephen.finn@tcd.ie. Address: Department of Histopathology and Morbid Anatomy, Sir Patrick Dun Translational Research Lab, St. James’s Hospital, Dublin, Ireland.

Email addresses and ORCID iDs:

Ezgi Oner: [ezgioner89@gmail.com](mailto:ezgioner89@gmail.com) (<https://orcid.org/0000-0002-4684-8221>)

Mustafa Kotmakci: [mustkot@gmail.com](mailto:mustkot@gmail.com) (https://orcid.org/0000-0002-5593-8608)

Anne-Marie Baird: [bairda@tcd.ie](mailto:bairda@tcd.ie) (<https://orcid.org/0000-0001-7501-1159>)

Steven G. Gray: [sgray@stjames.ie](mailto:sgray@stjames.ie) (https://orcid.org/0000-0002-5850-6392)

Bilge Debelec Butuner: [bilge.debelec@ege.edu.tr](mailto:bilge.debelec@ege.edu.tr) (https://orcid.org/0000-0001-8112-9241)

Emir Bozkurt: [emrbzkrt@gmail.com](mailto:emrbzkrt@gmail.com) (https://orcid.org/0000-0003-4703-397X)

Ayse Gulten Kantarci: [gulten.kantarci@ege.edu.tr](mailto:gulten.kantarci@ege.edu.tr) (https://orcid.org/0000-0001-8813-5353)

Stephen P. Finn: [stephen.finn@tcd.ie](mailto:stephen.finn@tcd.ie) (https://orcid.org/0000-0002-8628-5814)


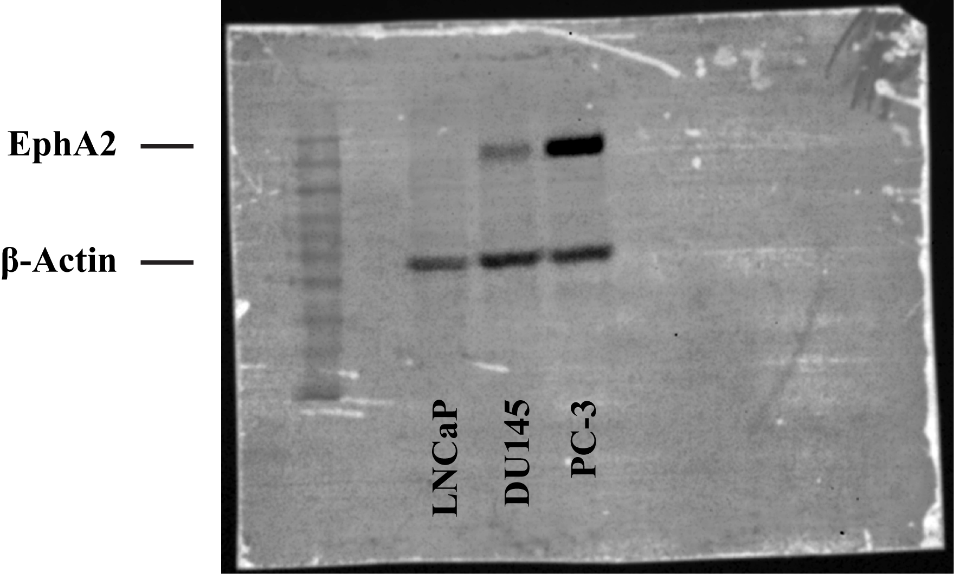


**Fig. S1.** Representative Western blot image showing the basal expression levels of EphA2 protein in LNCaP, DU145, and PC-3 prostate cancer cells.

β-actin was used as a loading control. Each well was loaded with 50 µg of total protein. Marker bands: 175, 130, 95, 70, 62, 51, 42, 29, 22, 14 kDa.

**
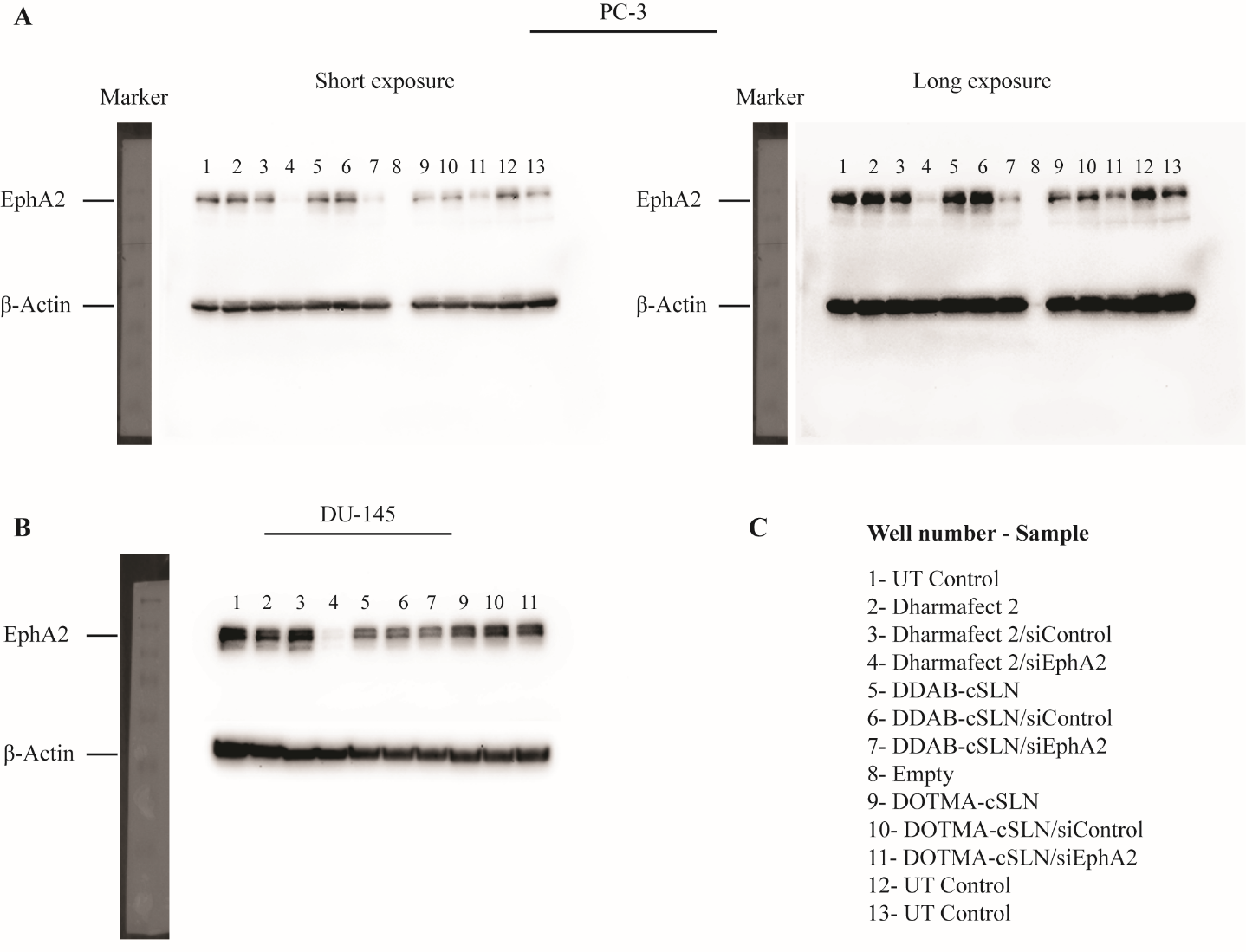
**

**Fig. S2.** Full scans of Western blot images corresponding to Fig. 6c, d. Marker bands: 250, 150, 100 75, 50, 37, 25, 20 kDa.


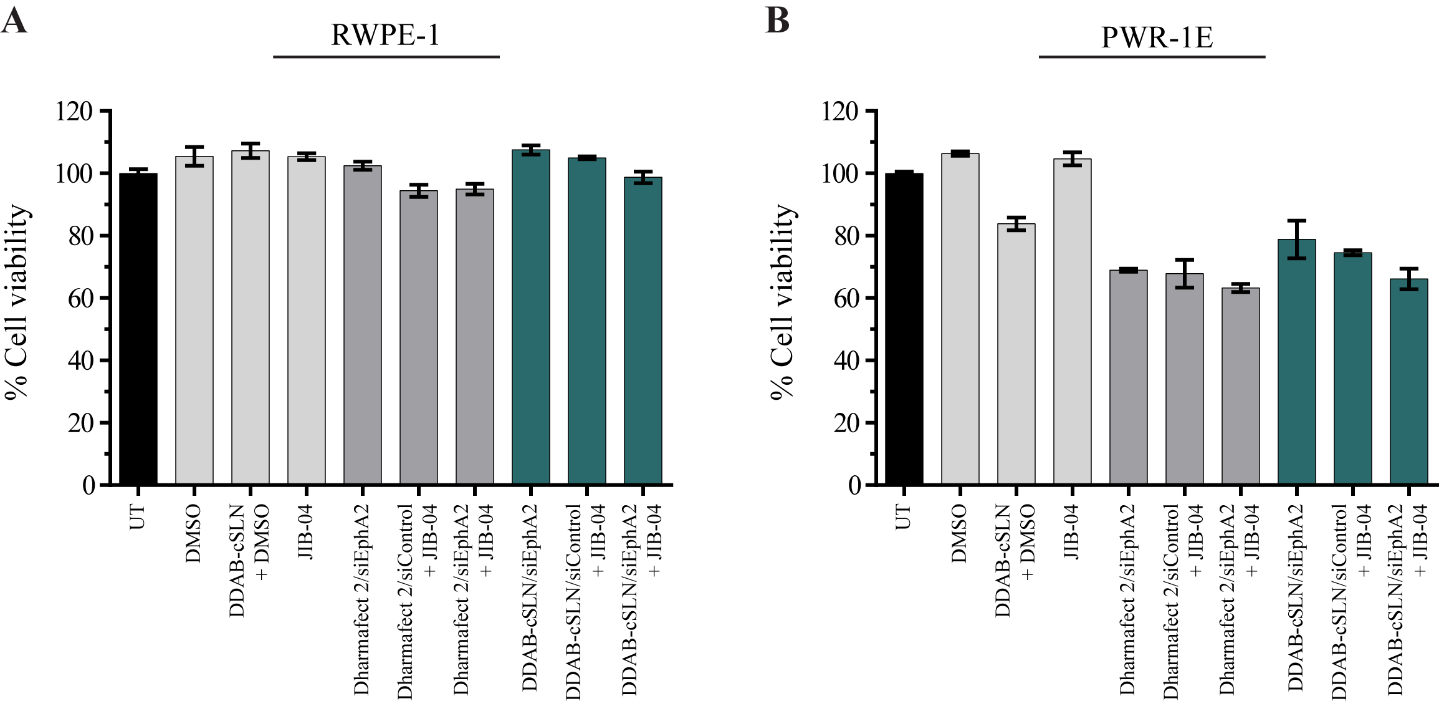


**Fig. S3.** The effect of siEphA2 complexes co-administered with JIB-04 on cell viability in RWPE-1 (**A**) and PWR-1E (**B**) normal human prostate epithelial cell lines (48 h). Data are given as mean ± SD of three measurements.

**
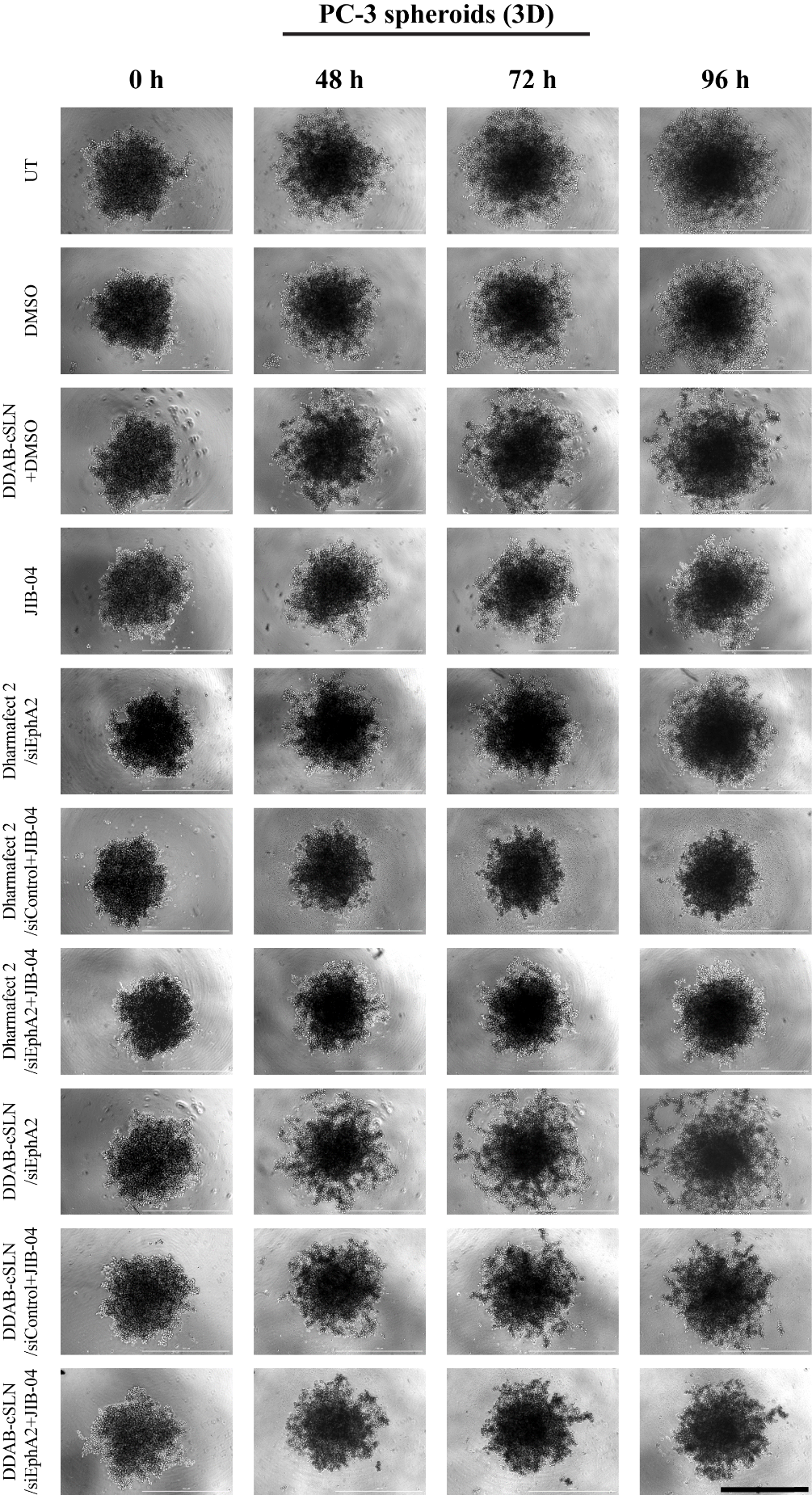
**

**Fig. S4.** The morphological changes in PC-3 spheroids after co-treatment of siEphA2 complexes with JIB-04. Scale bar, 1000 µm.


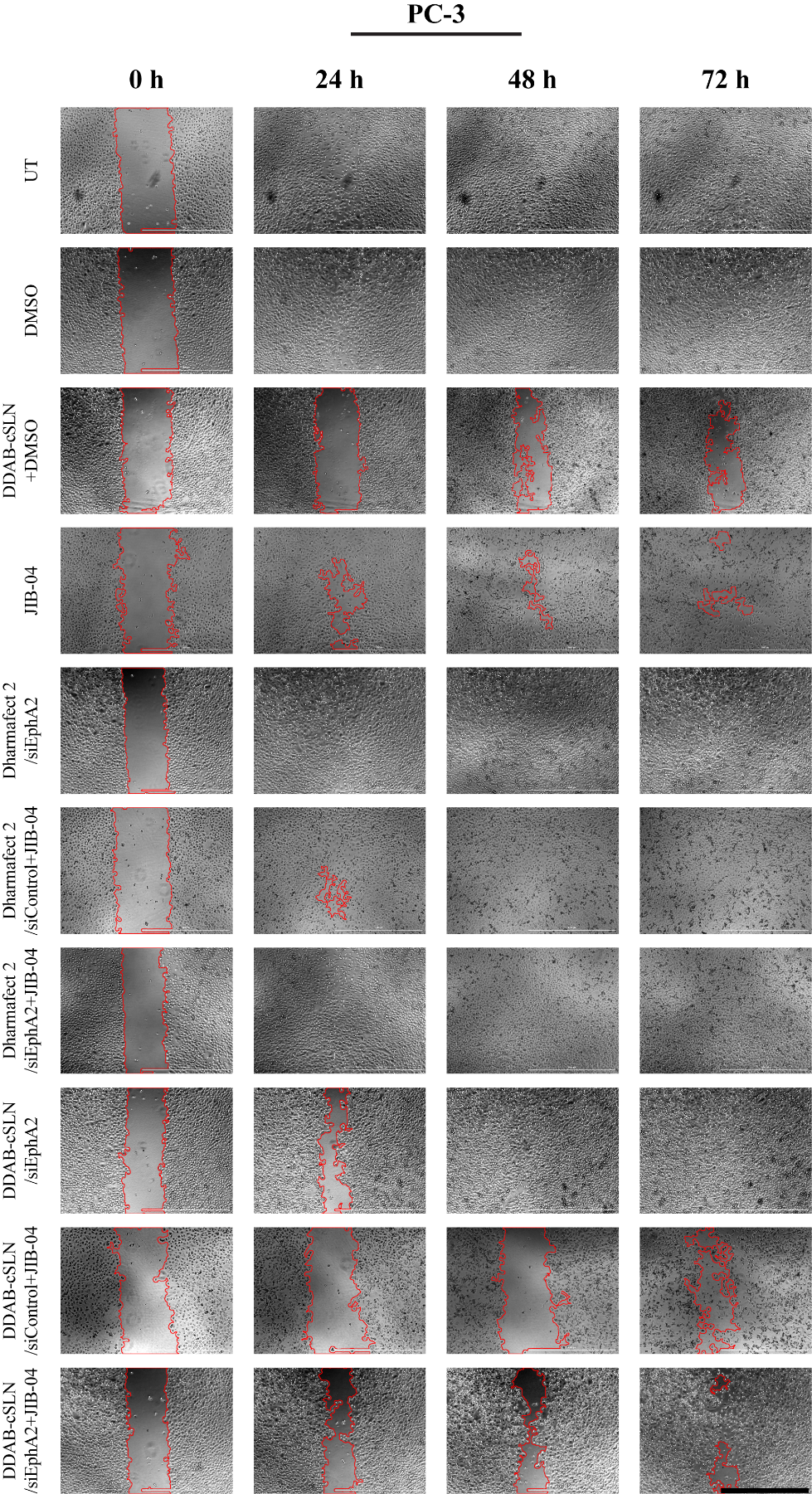


**Fig. S5.** Representative phase-contrast images of PC-3 cells showing the changes in scratch area in response to treatments after 0 h, 24 h, 48 h, and 72 h. *MRI Wound Healing Tool* plugin of ImageJ was used to automatically draw the scratch area. Scale bar, 1000 µm.
